# Brain-derived neurotrophic factor in cerebrospinal fluid and plasma is not a biomarker for Huntington’s disease

**DOI:** 10.1038/s41598-021-83000-x

**Published:** 2021-02-10

**Authors:** Zhen-Yi Andy Ou, Lauren M. Byrne, Filipe B. Rodrigues, Rosanna Tortelli, Eileanoir B. Johnson, Martha S. Foiani, Marzena Arridge, Enrico De Vita, Rachael I. Scahill, Amanda Heslegrave, Henrik Zetterberg, Edward J. Wild

**Affiliations:** 1 UCL Huntington’s Disease Centre, UCL Queen Square Institute of Neurology, University College London, London, WC1N 3BG UK; 2 UK Dementia Research Institute at UCL, London, WC1E 6BT UK; 3 Department of Neurodegenerative Disease, UCL Institute of Neurology, London, WC1N 3BG UK; 4 Lysholm Department of Neuroradiology, National Hospital for Neurology and Neurosurgery, London, WC1N 3BG UK; 5 Department of Biomedical Engineering, School of Biomedical Engineering and Imaging Sciences, King’s College London, London, SE1 7EH UK; 6 Department of Psychiatry and Neurochemistry, Institute of Neuroscience and Physiology, The Sahlgrenska Academy at the University of Gothenburg, 431 80 Mölndal, Sweden; 7 Clinical Neurochemistry Laboratory, Sahlgrenska University Hospital, 431 80 Mölndal, Sweden

**Keywords:** Neurotrophic factors, Huntington's disease, Biomarkers

## Abstract

Brain-derived neurotrophic factor (BDNF) is implicated in the survival of striatal neurons. BDNF function is reduced in Huntington’s disease (HD), possibly because mutant huntingtin impairs its cortico-striatal transport, contributing to striatal neurodegeneration. The BDNF trophic pathway is a therapeutic target, and blood BDNF has been suggested as a potential biomarker for HD, but BDNF has not been quantified in cerebrospinal fluid (CSF) in HD. We quantified BDNF in CSF and plasma in the HD-CSF cohort (20 pre-manifest and 40 manifest HD mutation carriers and 20 age and gender-matched controls) using conventional ELISAs and an ultra-sensitive immunoassay. BDNF concentration was below the limit of detection of the conventional ELISAs, raising doubt about previous CSF reports in neurodegeneration. Using the ultra-sensitive method, BDNF concentration was quantifiable in all samples but did not differ between controls and HD mutation carriers in CSF or plasma, was not associated with clinical scores or MRI brain volumetric measures, and had poor ability to discriminate controls from HD mutation carriers, and premanifest from manifest HD. We conclude that BDNF in CSF and plasma is unlikely to be a biomarker of HD progression and urge caution in interpreting studies where conventional ELISA was used to quantify CSF BDNF.

## Introduction

Huntington’s disease (HD) is a fatal neurodegenerative disorder caused by a mutation in the gene encoding mutant huntingtin protein (mHTT). It causes behavioural, cognitive, and motor dysfunctions and no disease-modifying treatment has yet demonstrated efficacy^1^.

In HD, the selective vulnerability and degeneration of striatal neurons may be caused by the depletion of brain-derived neurotrophic factor (BDNF)^2^. BDNF is a growth factor implicated in neuronal survival, development, and synaptic plasticity. HD post-mortem brain tissue has been shown to have reduced BDNF^3^. Murine studies demonstrate that the absence of BDNF leads to early striatal neuronal death and HD phenotypes^4,5^. BDNF concentration is lower in the striatum of R6/1 and zQ175 mice compared to the wild-type counterparts^6,7^. This lowered concentration may have been influenced by a wild-type huntingtin-mediated loss of function mechanism. Wild-type huntingtin regulates the transport of BDNF and indirectly promotes the genetic expression of BDNF^8,9^ and BDNF overexpression produced phenotypic recovery in a murine model^10^. BDNF and its trophic pathway are targets for therapeutic development^11^.

BDNF concentration in cerebrospinal fluid (CSF) or blood could be an accessible means of quantifying dysfunction of this pathogenic pathway, could be a useful monitoring or prognosis biomarker, and could elucidate target engagement by therapeutics expected to restore the pathway. BDNF in these fluids has been studied in several neurodegenerative diseases: patients with Parkinson’s and Alzheimer’s disease (AD) have reduced serum BDNF levels^12,13^, and restoration of BDNF may ameliorate behavioural deficits and neuronal loss in AD models^12^. Similarly, BDNF deficiency in serum has been observed in HD patients^14^.

However, BDNF is stored in platelets^15^ so its concentration in blood-derived products, such as plasma and serum, may not be an accurate reflection of the CNS due to platelet activation and degranulation. Moreover, all previous reports of BDNF levels in CSF in neurodegeneration have used conventional enzyme-linked immunosorbent assays (ELISAs) and have found concentrations below the linear range of the assay, raising doubt as to the validity and accuracy of the reported disease differences^16–18^.

To our knowledge, BDNF has not been successfully quantified in CSF in HD patients^19^ and no comparison between CSF and plasma levels has been performed using a suitable ultra-sensitive assay.

We therefore compared several immunoassays and quantified BDNF in blood and CSF from HD mutation carriers and healthy controls both cross-sectionally and over a 2-year longitudinal period to determine whether BDNF is a potential biomarker for HD.

## Materials and methods

### Study design

Participants (20 healthy controls, 20 premanifest HD (preHD), and 40 manifest HD) were recruited from the National Hospital for Neurology and Neurosurgery/University College London HD Multidisciplinary Clinic as part of a longitudinal CSF collection initiative (online protocol: https://doi.org/10.5522/04/11828448.v1)^20^. This was a single-site study affiliated with the HDClarity study(NCT02855476, http://hdclarity.net/) with added optional magnetic resonance imaging (MRI) and 6-week repeated sampling, and with a 24-month longitudinal follow-up. It was conducted according with the Declaration of Helsinki and was approved by the London–Camberwell St Giles Research Ethics Committee. All participants gave written informed consent.

Manifest HD participants were defined as adults having a Unified Huntington’s Disease Rating Scale (UHDRS) diagnostic confidence level (DCL) of 4 and HTT CAG repeat count ≥ 36. PreHD participants had CAG ≥ 40 and DCL < 4. Healthy controls were age- and gender-matched to gene expansion carriers, mostly spouses or gene-negative siblings of HD gene expansion carriers and with no neurological signs or symptoms. Manifest HD participants were staged according to UHDRS total functional capacity^21^.

### Study procedures

The inclusion and exclusion criteria, clinical assessments, blood and CSF collection and processing, and MRI acquisition and processing are described in the online protocol and previous reports^20,22^. Briefly, participants underwent clinical assessments including the UHDRS subscales. Blood samples were collected into lithium heparin tubes after an overnight fast and were centrifuged at 1300 g for 10 min at 4 °C to produce normal plasma (as opposed to platelet-poor plasma)^23^ and aliquoted on ice and stored at − 80 °C using a standardised protocol. T1-weighted MRI data were acquired on a 3 T Siemens Prisma scanner as previously described^20^. Brain volume change was expressed as change per year and adjusted to total intracranial volume.

### Assay techniques

#### Immunoassays comparison

BDNF was quantified in duplicate in 20 CSF and serum samples from deidentified individuals from a clinical neurochemistry laboratory to compare the sensitivity of commercially available immunoassays: BDNF Emax ImmunoAssay System (Promega, USA), Human BDNF ELISA (Sigma-Aldrich, USA), and Single molecule array (Simoa) Human BDNF Discovery Kit (Quanterix, USA). POLARstar Omega (BMG Labtech, Germany) was used to measure the fluorescent products in the standard ELISAs, whilst an HD-1 Analyzer (Quanterix, USA) was used for the Simoa kit. For Promega and Sigma-Aldrich ELISAs, the limit of detection (LOD) and quantification (LOQ) were calculated from raw absorbance data.

#### The HD-CSF cohort

Plasma and CSF BDNF from the HD-CSF cohort were quantified in duplicate using the Simoa kit on an HD-1 Analyzer according to manufacturer guidance. Plasma samples were measured at 1:640 and CSF at 1:4 dilution. The lower LOQ and lower LOD were 0.0293 pg/mL and 0.0026 pg/mL, respectively. All measurements were above both. The intra-assay coefficients of variation (CV; calculated as the mean of the CVs for each sample’s duplicate measurements) was 8.30% for CSF (n = 84) and 3.26% for plasma (n = 91). The inter-assay CV (calculated as the mean of the CVs for analogous spiked positive controls provided by the manufacturer and used in each well plate) was 4.39% for CSF (n = 2) and 2.74% for plasma (n = 4). Four (4.8%) CSF samples were only measured once. Quantification was blinded to disease status.

#### Statistical methods

For the HD-CSF cohort, BDNF distribution was assessed for normality, and plasma and CSF BDNF values underwent square root and inverse square transformation, respectively.

General linear models and Pearson’s chi-squared or Fisher’s exact test were performed to assess intergroup differences at baseline and at the 24-month follow-up characteristics.

Potential confounders including age, gender, body mass index (BMI), medication, serum platelet counts, and CSF blood contamination (hemoglobin and erythrocytes), storage duration, and short problem behaviour assessment (PBA-s) subscores were examined through general linear models, Pearson’s correlations or ordinal logistic regressions. Those expected to have a relevant impact on BDNF, either based on reports from the literature, or directly seen in our sample, were included as covariates for subsequent models^15,24–27^. All included gender, BMI, anti-depressant, anti-psychotic medication, and age. For plasma we also included sample storage duration and platelet count. For CSF we also included CSF erythrocyte count. As a relevant contributor to HD natural history, CAG repeat length was included in the models.

BDNF intergroup differences were investigated with general linear models. Association in HD mutation carriers with clinical and imaging measures were investigated with Pearson’s partial correlations with bootstrapped bias corrected and accelerated 95% confidence intervals (95%CI). Receiver operating characteristic (ROC) curves were drawn for plasma and CSF BDNF.

To investigate short term stability, two-way mixed effects models intraclass correlations (ICC) were performed between the baseline and optional 6-week samples.

Annualised rates of change in BDNF and clinical measures were calculated by subtracting baseline from follow-up value and dividing by time between samplings. Intergroup differences and associations in HD mutation carriers with clinical and imaging measures were investigated as above.

To study BDNF longitudinal trajectories, we used mixed effects models with age and potential confounders as fixed effects, and random effects for participant (intercept) and age (slope), generated independently for controls and mutation carriers.

All analyses were performed with Stata 15.1 (StataCorp). The significance level was defined as *p* value < 0.05. No methods for multiplicity correction were used, so any statistically significant outcomes would need to be considered in the context of related statistical tests.

## Results

### Immunoassay comparison

Serum BDNF levels in 20 test samples were in the linear range for tested assays. However, CSF levels were below the LOD in all 20 samples on the Promega assay, while 16 (80%) out of 20 samples were between the LOD and the LOQ on the Sigma-Aldrich assay. In plasma, all tested assays were correlated (Simoa and Promega: r = 0.9200, *p* < 0.0001; Simoa and Sigma-Aldrich: r = 0.9428, *p* < 0.0001; Promega and Sigma-Aldrich: r = 0.9175, *p* < 0.0001). In CSF, Simoa and Sigma-Aldrich showed a weaker association (r = 0.4269, *p* = 0.0991). The Simoa assay quantified BDNF concentration in blood and CSF within the linear range, and above the LOD and LOQ in all samples. This assay was therefore used for all subsequent analyses in both biofluids.

### Cross-sectional analysis of baseline

#### Demographic characteristics and confounding variables

Demographic and clinical characteristics are summarised in Table 1. The control group was matched to the HD mutation carriers as a whole but was older on average than preHD, which in turn was younger than the HD group. The preHD group had a lower mean BMI than the controls, but did not differ from HD. More manifest HD participants were on medication, including anti-depressants and anti-psychotics.

**Table 1:** Baseline characteristics of the HD-CSF cohort. Presented p values are not adjusted for multiple comparisons. Results were replicated at follow-up. Brain volumes are percentages of total intracranial volume. Values are mean ± SD, except where it stated otherwise. Manifest HD, manifest HD mutation carriers; Premanifest HD/preHD, premanifest HD mutation carriers; SD, standard deviation; ANOVA, analysis of variance; PBA-s, Problem Behaviours Assessment-short version; CSF, cerebrospinal fluid; BMI, body-mass index; cUHDRS, composite Unified Huntington’s Disease Ratings Scale; UHDRS, Unified Huntington’s Disease Ratings Scale; N/A, not applicable.

	Control	Premanifest HD	Manifest HD	ANOVA (*p* value)	Control versus PreHD (*p* value)	PreHD versus HD (*p *value)
N	20	20	37	N/A	N/A	N/A
Males *n *(%)	10 (50)	10 (50)	19 (51)	0.993	1.000	0.922
Age (years)	50.7 ± 11.0	42.4 ± 11.0	56.4 ± 9.5	< 0.0001	0.013	< 0.0001
BMI (kg/m^2^)	29.0 ± 7.9	25.1 ± 3.0	24.8 ± 5.0	0.020	0.027	0.859
CAG repeats	N/A	42.4 ± 1.6	42.7 ± 2.3	N/A	N/A	0.207
Disease burden score	N/A	267.1 ± 61.9	396.4 ± 97.5	N/A	N/A	< 0.0001
cUHDRS	17.4 ± 1.5	18.0 ± 1.1	10.4 ± 3.6	< 0.0001	0.496	< 0.0001
UHDRS total functional capacity	13.0 ± 0.0	13.0 ± 0.0	9.4 ± 2.8	< 0.0001	1.000	< 0.0001
UHDRS total motor score	2.4 ± 2.4	2.8 ± 2.8	37.8 ± 19.7	< 0.0001	0.919	< 0.0001
Symbol digit modalities test	50.9 ± 10.4	55.6 ± 9.3	26.8 ± 12.8	< 0.0001	0.199	< 0.0001
Stroop word reading	100.2 ± 17.4	105.1 ± 11.8	59.6 ± 24.0	< 0.0001	0.438	< 0.0001
Stroop color naming	75.8 ± 13.1	81.3 ± 10.1	46.0 ± 16.9	< 0.0001	0.233	< 0.0001
Verbal fluency—categorical	24.3 ± 4.1	23.3 ± 3.4	14.3 ± 5.9	< 0.0001	0.522	< 0.0001
Whole brain volume	80.1 ± 3.7	79.5 ± 3.3	70.4 ± 4.9	< 0.0001	0.716	< 0.0001
Gray matter volume	47.3 ± 3.5	47.5 ± 3.1	39.6 ± 4.3	< 0.0001	0.881	< 0.0001
White matter volume	29.4 ± 2.2	28.8 ± 1.9	25.6 ± 2.4	< 0.0001	0.443	< 0.0001
Caudate volume	0.455 ± 0.077	0.409 ± 0.074	0.286 ± 0.098	< 0.0001	0.149	< 0.0001
Any medication *n *(%)^a^	15 (75)	15 (75)	36 (97)	0.010	1.000	0.017
Anti-depressant *n *(%)^a^	3 (15)	4 (20)	30 (81)	< 0.001	1.000	< 0.001
Anti-psychotic *n *(%)^a^	0 (0)	0 (0)	15 (41)	< 0.001	1.000	0.001
Anti-epileptic *n *(%)^a^	0 (0)	0 (0)	2 (5)	0.494	1.000	0.536
Sleep-related drugs *n *(%)^a^	1 (5)	2 (10)	5 (14)	0.727	1.000	1.000
PBA-s depression severity^b^	0.7 ± 0.9	0.6 ± 1.1	0.9 ± 1.2	0.501	0.587	0.249
PBA-s suicidal ideation severity^b^	0.0 ± 0.0	0.2 ± 0.5	0.3 ± 0.7	0.999	0.995	0.967
PBA-s anxiety severity^b^	0.9 ± 1.1	1.3 ± 1.2	1.2 ± 1.0	0.506	0.273	0.771
PBA-s apathy severity^b^	0.2 ± 0.5	0.3 ± 0.9	0.9 ± 1.1	0.005	0.975	0.014
PBA-s obsessive–compulsive behaviours severity^b^	0.1 ± 0.4	0.3 ± 0.8	0.2 ± 0.6	0.597	0.312	0.626
PBA-s delusions severity^b^	0.0 ± 0.0	0.0 ± 0.0	0.1 ± 0.5	1.000	1.000	0.998
Platelet counts in blood (10^9^/L)^c^	236.0 [219, 260]	240.5 [212, 253.5]	269.0 [215, 301]	0.148	0.491	0.058
Erythrocytes counts in CSF (per uL)^c^	0.8 [0.0, 5.0]	0.8 [0.0, 5.0]	0.7 [0.0, 4.0]	0.443	0.884	0.262
Hemoglobin in CSF (ng/mL)	416.7 ± 634.2	475.9 ± 543.2	262.6 ± 243.0	0.200	0.683	0.096

^a^Fisher’s exact test.

^b^Ordinal logistic regression.

^c^Median [Q1, Q3].

Plasma and CSF BDNF did not differ by gender (Figure S1). The platelet count in blood did not differ between groups, but it was positively associated with plasma BDNF in HD mutation carriers (Figure S2). In the HD group, the average erythrocyte count was negatively correlated with CSF BDNF concentration (Figure S2).

#### Storage duration as a BDNF confounder in plasma but not CSF

**Figure 1:**
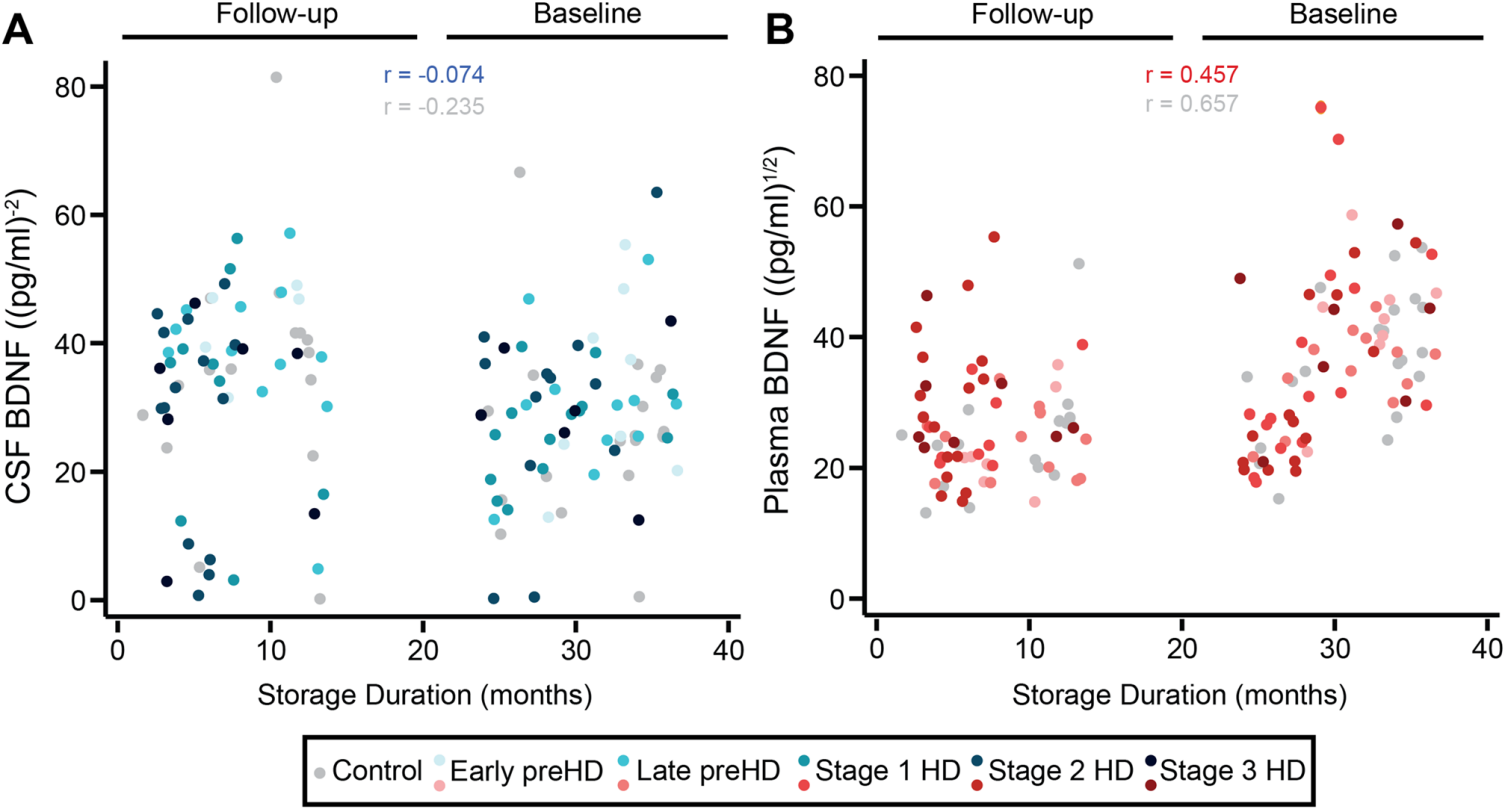
Association between biofluid storage duration and BDNF concentrations. Correlation between the storage duration in months and CSF BDNF (**A**) and plasma BDNF (**B**). CSF BDNF values were inverse square transformed and plasma BDNF were square root transformed. CSF, cerebrospinal fluid; HD, manifest HD mutation carriers; PreHD, premanifest HD mutation carriers; BDNF, brain-derived neurotrophic factor.

BDNF levels in baseline plasma samples were substantially higher than in the 24-month follow-up samples (Fig. 1). This was not seen in CSF, suggesting the possibility that BDNF levels were rising artefactually over time in frozen plasma, perhaps due to leakage from small numbers of residual platelets or degradation of pro-BDNF to BDNF. In keeping with this, the time spent in frozen storage was positively associated with plasma BDNF but not CSF BDNF, in controls and HD mutation carriers (Fig. 1). Hence, plasma BDNF analyses were additionally adjusted for sample storage duration.

#### BDNF levels in Huntington’s disease

**Figure 2:**
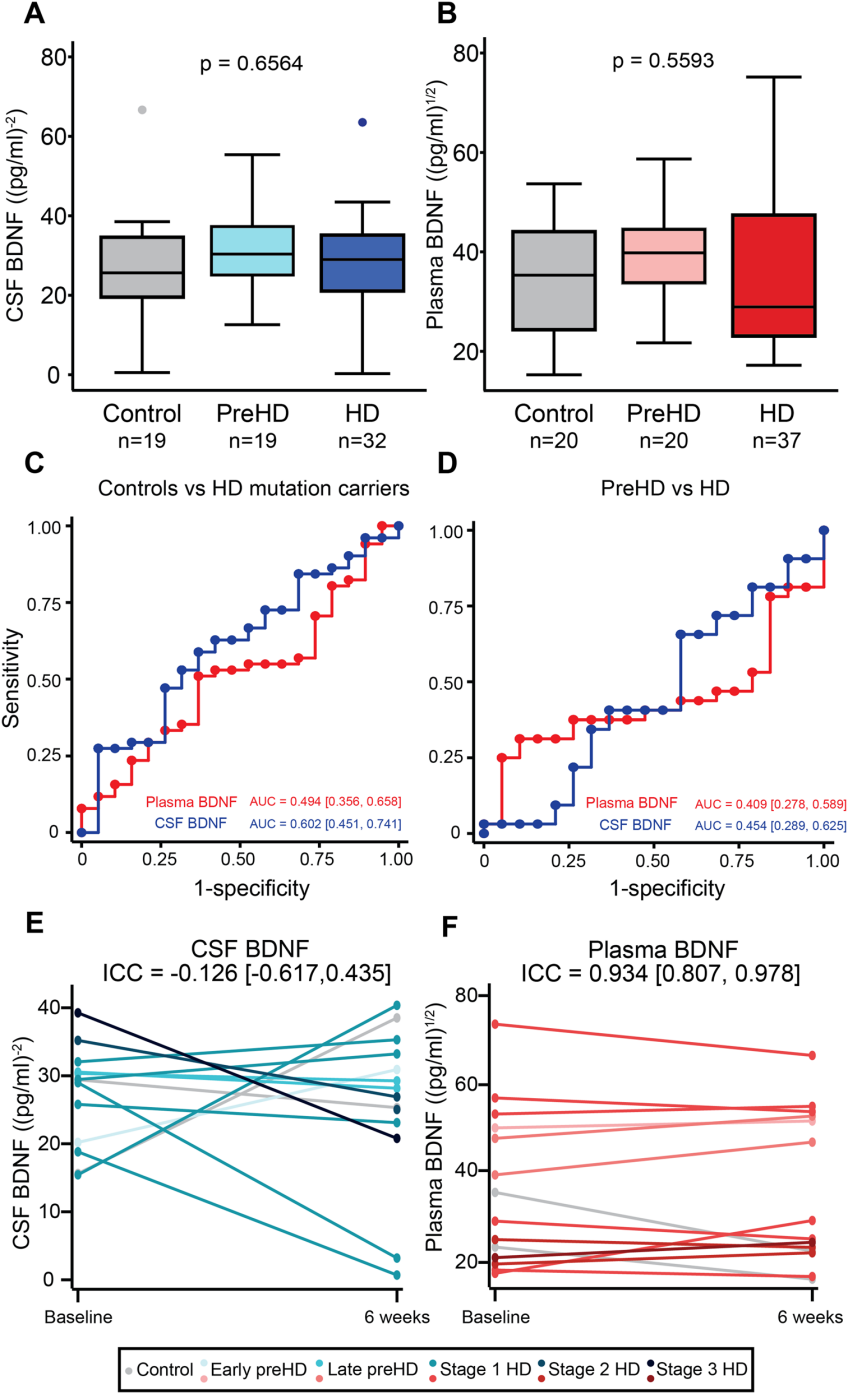
Baseline BDNF plots. Concentration of BDNF in CSF (**A**) and plasma (**B**) in healthy controls, premanifest HD (PreHD), and manifest HD (HD) patients. Comparison were generated with general linear models and were adjusted for CSF (gender, BMI, anti-depressant, anti-psychotic medication, age, CAG repeats, and erythrocyte count) or plasma covariates (gender, BMI, anti-depressant, anti-psychotic medication, age, CAG repeats, sample storage duration, and platelet count). ROC curves for (**C**) discrimination between healthy control (n = 19) and HD mutation carriers (n = 51) and (**D**) discrimination between premanifest HD (n = 19) and HD patients (n = 32). Results (**A**–**D**) were replicated at follow-up. Stability of BDNF in CSF (**E**) and plasma (**F**) over approximately 6 weeks was assessed with two-way mixed effects models intraclass correlation (ICC). Each line described the same participant (n = 13 in CSF and n = 14 in plasma) between the baseline and optional 6-week repeated sampling. CSF BDNF values were inverse square transformed and plasma BDNF were square root transformed. AUC, area under the curve; CSF, cerebrospinal fluid; preHD, premanifest HD mutation carriers; HD, manifest HD mutation carriers; BDNF, brain-derived neurotrophic factor.

Neither plasma nor CSF BDNF differed significantly between groups (Fig. 2). There were no associations between plasma or CSF BDNF and clinical measures, with the sole exception of plasma BDNF which was weakly positively associated with Verbal Fluency—Categorical (r = 0.261, *p* = 0.015; Figure S3). There were no associations between BDNF and imaging measures, except a weak positive association between CSF BDNF and white matter volume (r = 0.306, *p* = 0.032; Figure S4). It is of note that no correction for multiplicity was applied.

Plasma and CSF BDNF showed poor ability to distinguish controls from HD mutation carriers (plasma AUC = 0.494, CSF AUC = 0.602; Fig. 2C) and preHD from HD (plasma AUC 0.409, CSF AUC = 0.454; Fig. 2D).

CSF BDNF concentrations significantly fluctuated between the baseline and 6-week follow-up period (CSF ICC = − 0.126, Fig. 2E). Plasma concentrations were stable (ICC = 0.934, Fig. 2F).

### Longitudinal analysis

**Figure 3:**
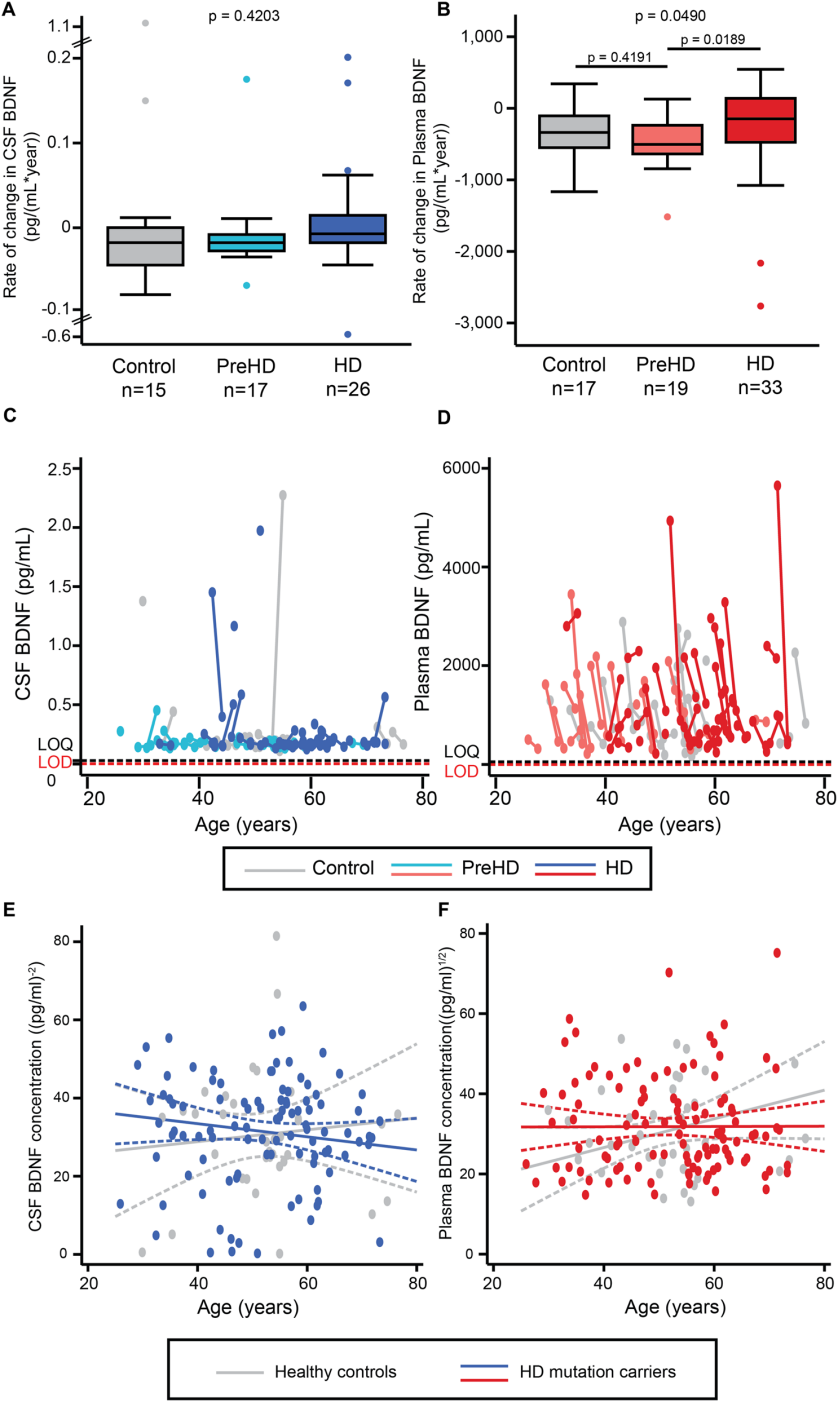
Longitudinal analyses in CSF and plasma. Rate of change in the concentration of BDNF in CSF (**A**) and plasma (**B**) in healthy controls, premanifest HD (PreHD), and manifest HD (HD) patients. Comparison were generated with general linear models. Relationship between raw CSF (**C**) and plasma BDNF values (**D**) and age in years. The limit of quantification (LOQ) and limit of detection (LOD) of the SIMOA assay are indicated. Longitudinal trajectories were studied with mixed effect models, which are adjusted for covariates (**E**,**F**). CSF, cerebrospinal fluid; BDNF, brain-derived neurotrophic factor; HD, manifest HD mutation carriers, PreHD, premanifest HD mutation carriers.

The rate of change in CSF BDNF did not differ between any HD stages (Fig. 3A,B). Longitudinal evaluation of plasma BDNF was limited by its apparent tendency to rise in freezer storage, producing spuriously higher values for baseline samples (Fig. 3C,D), but using the mixed effect model with adjustment for confounders, CSF and plasma BDNF did not change over time in controls or HD mutation carriers (Fig. 3E,F). Overall, baseline BDNF did not predict subsequent change in clinical measures (Figure S5), nor did the rates of change in BDNF (Figure S6).

## Discussion

The conventional ELISAs we tested lacked the sensitivity required to accurately quantify CSF BDNF. With an ultra-sensitive assay, BDNF levels were measurable in both CSF and plasma but did not reflect HD or its progression and were not associated with the examined clinical and imaging measures.

It was notable that the ELISA assays used in previous reports of altered CSF BDNF in neurological disease appear insufficiently sensitive for the purpose. This emphasizes the importance of ensuring the analyte in question lies in the assay’s linear range and urges caution when interpreting previous reports of CSF BDNF as a disease biomarker. To our knowledge, this is therefore the first report of BDNF levels in human CSF using an assay capable of reliably quantifying them.

Unexpectedly, we also found that the storage duration is the major influence upon BDNF level in plasma but not CSF. This diminishes the utility of stored frozen samples for BDNF quantification in plasma and suggests time in storage should be controlled for in future biomarker studies. BDNF is known to be enriched in platelets, and our centrifugation procedure was designed to produce normal as opposed to platelet-poor plasma. The latter may show less influence from platelet-derived BDNF and therefore be a better surrogate for brain BDNF^23^.

BDNF had no properties suggesting it is a biomarker for HD progression in CSF or plasma. Its level did not differ significantly between controls and HD mutation carriers, or between preHD and HD groups. It had poor classification ability in our ROC analysis and lacked associations with clinical and MRI brain volumetric measures. In the context of a clear lack of groupwise differences, the small number of associations seen among many tests is likely a chance finding and not of biological significance. Neither baseline BDNF level, nor its rate of change, were prognostic for subsequent change in clinical or imaging outcomes. We therefore conclude that BDNF in CSF or plasma is unlikely to be a valid biomarker of HD, in the ‘diagnostic’, ‘monitoring’ or ‘prognostic’ categories according to the FDA’s BEST categorisation.

These negative findings contrast starkly with the performance of other CSF and plasma analytes, most notably neurofilament light and mutant huntingtin proteins, which do differ between groups, rise with progression, and predict subsequent clinical and imaging change^20,22,28^.

It should be noted that the absence of detectable changes in CSF or plasma does not exclude an important role for BDNF or its trophic pathway in the pathogenesis of HD. The decrease of BDNF might be very region-specific and therefore overwhelmed by generally unaltered BDNF in other regions. Several studies found reduced BDNF in striatum but not cortex in HD models^3,7,9^. Moreover, if striatal BDNF loss is significant, other gene promotors of the BDNF gene that are not directly modulated by HTT could possibly compensate for the mHTT-induced deficit of BDNF in the cortex^29^.

In the present study, we did not investigate BDNF isoforms, including its precursor, pro-BDNF. Studying the isoforms of BDNF, including pro-BDNF, could still reveal disease-related changes. Unlike some previous reports, we studied plasma rather than serum, but since plasma is prepared by centrifugation rather than clotting, we reasoned that it was less likely to contain artefactual signals from platelets.

Finally, it is important to note that even though we found BDNF to lack utility as a biomarker of HD in the natural history setting, it is still theoretically possible that a therapeutic intervention could produce detectable and meaningful changes in CSF or plasma BDNF. Our findings may be of value for the design of such trials in which BDNF would serve as a pharmacodynamic biomarker.

## Supplementary Information

Supplementary Information.

## Data Availability

The data that support the findings of this study are available from the corresponding author, EJW, upon reasonable request.
